# Reply to Jobling, “Ectopic Expression of the *ydaS* and *ydaT* Genes of the Cryptic Prophage Rac of *Escherichia coli* K-12 May Be Toxic but Do They Really Encode Toxins?: a Case for Using Genetic Context To Understand Function”

**DOI:** 10.1128/mSphere.00177-18

**Published:** 2018-04-25

**Authors:** Aswin Sai Narain Seshasayee

**Affiliations:** a National Centre for Biological Sciences, Tata Institute of Fundamental Research, GKVK, Bangalore, India; University of Iowa

**Keywords:** prophage, toxicity, transcription factor

## REPLY

My group thanks Dr. Jobling for his insightful letter (1) that argues for a regulatory role for YdaS/T, encoded in the *rac* prophage of Escherichia coli. This argument is brought up as a critique of our suggestion that YdaS/T may be a toxin. We are glad that our work has generated interest in the community and take this opportunity to present the background to our study, reported in two papers, and also summarize our findings to remove any ambiguity that our papers might have caused in the minds of our readers.

Dr. Jobling is correct in pointing out that our paper focuses “solely on the toxicity of these two genes at the expense of any insight into their toxicity.” We also appreciate Dr. Jobling’s perspective, which arises from his own recent work on toxins encoded in full-length *rac* prophages encoded in pathogenic E. coli (2). Indeed, the fact that these proteins share homology—albeit limited at the sequence level—with lambda Cro and cII would throw insight into potential mechanisms of action. For example, it was shown that overexpression of cII causes toxicity by perturbing replication (3), a work that Dr. Jobling quotes. Similar analysis of the mechanism of toxicity of YdaS/T is certainly of interest, but that was not quite the objective of our study and is unlikely to be pursued by our lab in future.

Early in 2011, we were interested in knowing whether horizontally acquired transcription factors in E. coli K-12 had any effect on transcription of core genes. By chance, we landed upon RacR, a prophage-encoded putative transcription factor with weak homology to lambda cI. We realized that we could not delete the *racR* gene, and neither could the Keio collection effort (4). Our objective therefore was to find out what RacR’s targets were and how its function as a transcription factor might contribute to its essentiality to the E. coli cell. Our objective here was not to elucidate the effect of RacR or its targets on the life cycle of the *rac* prophage in its intact or defective form. Over the next few years, we gathered evidence to support the view that RacR represses *ydaS/T* and that this activity might be responsible for its essentiality. We summarize key pieces of evidence gathered from our two papers below:

(i) Deletions of *ydaS* and *ydaT* fully suppress the lethality of *ΔracR*. *ydaS* and *ydaT* are not expressed under normal growth conditions. Across a variety of conditions sampled in publicly available transcriptome sequencing (RNA-seq) data sets, *ydaS/T* is expressed to a level comparable to that of the well-established silent *bgl* operon (5).
(ii) Ectopic expression of *ydaS* and *ydaT* from arabinose-inducible pBAD vectors is toxic to the cell (5). We were indeed worried about expression from a plasmid and artifacts that might arise from this construct. At this point, we established a collaboration with Devashish Rath, who had tools based on clustered regularly interspaced short palindromic repeat(s) (CRISPR) to downregulate *racR* and study its effect on *ydaS/T* expression.
(iii) CRISPR-mediated downregulation of *racR* increased the expression of *ydaS/T* (6).
(iv) Downregulation of *racR* also resulted in decreased fitness. However, downregulation of *racR* did not affect fitness in the *ΔydaS-ydaT* background (6).
(v) RacR binds to the intergenic region between *racR* and *ydaS*, as measured by chromatin immunoprecipitation-quantitative PCR (ChIP-qPCR) and biochemical thermal shift assays and electrophoretic mobility shift assays (EMSAs) (5).
(vi) The *rac* prophage is mosaic across E. coli strains as shown by bioinformatic analysis of publicly available E. coli genomes. Even the occurrence of *racR* and *ydaS/T* is patchy. However, *ydaS/T* is present only in strains in which *racR* is also present (5).

Together, these show that the repression of *ydaS/T* by RacR is largely responsible for its essentiality to its host E. coli K-12 cell. These indicate that YdaS/T expression—from a plasmid, or from the chromosome when *racR* is downregulated—is toxic to the cell. The mechanism of action of YdaS and YdaT is not known, and their similarities to Cro and cII might give some pointers in that direction.

These results do not in any way argue against the potential role of these proteins as regulatory switches within the phage; addressing this issue was always beyond the scope of our work. It is indeed possible and quite likely that the toxic activity of these proteins against E. coli is incidental and a by-product of their regulatory activity within the *rac* prophage.
